# Identification and comparative proteomic study of quail and duck egg white protein using 2-dimensional gel electrophoresis and matrix-assisted laser desorption/ionization time-of-flight tandem mass spectrometry analysis

**DOI:** 10.3382/ps/pew033

**Published:** 2016-03-08

**Authors:** Shan Hu, Ning Qiu, Yaping Liu, Hongyan Zhao, Dan Gao, Rui Song, Meihu Ma

**Affiliations:** National Research and Development Center for Egg Processing, College of Food Science and Technology, Huazhong Agricultural University, Wuhan, Hubei 430070, P. R. China

**Keywords:** avian, egg white protein, proteomic, 2-DE, MALDI-TOF MS/MS

## Abstract

A proteomic study of egg white proteins from 2 major poultry species, namely quail (*Coturnix coturnix*) and duck (*Anas platyrhynchos*), was performed with comparison to those of chicken (*Gallus gallus*) through 2-dimensional polyacrylamide gel electrophoresis (2-DE) analysis. By using matrix-assisted laser desorption/ionization time-of-flight tandem mass spectrometry (MALDI-TOF MS/MS), 29 protein spots representing 10 different kinds of proteins as well as 17 protein spots designating 9 proteins were successfully identified in quail and duck egg white, respectively. This report suggested a closer relationship between quail and chicken egg white proteome patterns, whereas the duck egg white protein distribution on the 2-DE map was more distinct. In duck egg white, some well-known major proteins, such as ovomucoid, clusterin, extracellular fatty acid-binding protein precursor (ex-FABP), and prostaglandin D2 synthase (PG D2 synthase), were not detected, while two major protein spots identified as “deleted in malignant brain tumors 1” protein (DMBT1) and vitellogenin-2 were found specific to duck in the corresponding range on the 2-DE gel map. These interspecies diversities may be associated with the egg white protein functions in cell defense or regulating/supporting the embryonic development to adapt to the inhabiting environment or reproduction demand during long-term evolution. The findings of this work will give insight into the advantages involved in the application on egg white proteins from various egg sources, which may present novel beneficial properties in the food industry or related to human health.

## INTRODUCTION

Egg white protein is one of the most traditional raw materials for the food industry because of its foaming and gelling properties. Considering its biological functions in avian reproduction, egg white provides essential nutrients as well as protection against invading bacteria for embryo development. Egg whites from different avian origins could have various protein profiles according to evolution and adaptation under diverse environmental stresses. Chicken, quail, and duck egg are among the most consumed avian eggs worldwide. Former studies have revealed that the protein contents of lyophilized egg white from duck and quail were higher than that from chicken (Miguel et al., 2005). Additionally, duck and quail derived egg white proteins or peptides are proven to be very useful and could, in some cases, present certain advantages more than chicken egg white proteins. For example, duck ovostatin (ovomacroglobulin) is capable of inhibiting serine proteinase as well as metalloproteinase, while chicken ovostatin inhibits only metalloproteinase (Nagase et al., 1986). It was also reported that the reduced duck lysozyme has higher antimicrobial activity against *Salmonella enteritidis* than reduced chicken lysozyme (Hayakawa et al., 2006). Thus, interspecies variability may explain particular characteristics concerning technological, functional, and biological performance of egg proteins from different avian origins (Miguel et al., 2005). As egg is one of the foods that most frequently cause allergies, the variations of specific allergen activity in the various egg whites were widely investigated. Ovalbumin, ovomucoid, ovotransferrin, and lysozyme are 4 main allergens in egg white (Li et al., 2008). Anibarro et al. (2000) reported an unusual case of food allergy after consumption of eggs from duck and goose in an adult patient without hen egg allergy and demonstrated that ovalbumin was responsible for the allergy. Meanwhile, they suggested that the antigenic determinant of ovalbumin was specific to the Anseriformes order. So ascertainment of avian egg genuineness is an important topic, especially for safety reasons for different avian egg white allergic subjects (Anibarro et al., 2000). Moreover, these egg white protein variations among avian species suggested advantages in the use of proteins from different egg sources (Miguel et al., 2005). Thus, comparative studies on egg proteins from these poultry species are necessary to define the unique protein profile of each.

Various chicken egg white proteomic (Guerin-Dubiard et al., 2006; D'Ambrosio et al., 2008; Mann, 2007; Mann and Mann, 2011) and comparative proteomic (Omana et al., 2011; Qiu et al., 2012a; Qiu et al., 2012b; Wang et al., 2012; Liu et al., 2015; Qiu et al., 2013) studies have been performed during the last decade to gain a well-defined chicken egg white protein profile. Nevertheless, the egg white proteome profiles from other poultry species were less known. The phylogenetic analysis of the complete sequence of the Japanese quail (*Coturnix japonica*) mitochondrial genome with related species displayed a closer genetic relationship between quail and chicken than duck (Nishibori et al., 2002). Later, the duck (*Anas platyrhynchos*) genome was published in 2013, revealing a contractive immune-related gene repertoire distinct to that of chicken (Huang et al., 2013). These genomic data made it possible to compare the egg protein profiles from these 3 avian origins that were most economically important in the egg industry.

The aim of this work was to reveal and compare the proteomic pattern of the egg white proteins from 3 main poultry species by 2-dimensional gel electrophoresis (**2-DE**) and matrix-assisted laser desorption/ionization time-of-flight tandem mass spectrometry (**MALDI-TOF MS-MS**) analysis in order to investigate new potential egg white properties and functions.

## MATERIALS AND METHODS

### Egg White Sampling and Protein Extraction

The eggs of chicken, quail, and duck were collected within 2 to 3 days after laid from the Poultry Research Center farm of Huazhong Agricultural University and used in this study. Eggs were randomly selected for sampling. Three biological replicates and 2 technical replicates were performed during the following experiments for 2-DE analysis to reduce variations. Protein extraction was performed basically according to the previously described method (Qiu et al., 2012a). Briefly, the egg white was manually separated from the egg yolk and gently homogenized at 4 °C for 30 min with a magnetic stirrer. The egg white samples were centrifuged and resuspended 3 times. The protein content was quantified using a 2-DE Quant Kit (GE Healthcare, Piscataway, NJ).

### 2-Dimensional Gel Electrophoresis Analysis

The egg white proteins were analyzed by 2-DE analysis using the Ettan IPGphor 3 System (GE Healthcare, Piscataway, NJ, USA) for the first dimension isoelectric focusing (**IEF**) and the Ettan DALTSix System (GE Healthcare) to perform sodium dodecyl sulfate polyacrylamide gel electrophoresis (**SDS-PAGE**) in the second dimension as described previously (Qiu et al., 2012a). IEF was performed on DryStrip IPG strips of (24 cm; pH 4 to 7) with 125 μL (100 μg protein) of the protein sample in the rehydration buffer (Bio-Rad). IEF was performed on DryStrip IPG strips of (24 cm; pH 4 to 7) with 125 μL (100 μg protein) and was conducted at 20 °C using a gradient mode, step 1: 300 V for 0.5 h, step 2: 700 V for 0.5 h, step 3: 1500 V for 1.5 h, step 4: 9000 V for 3 h, step 5: 9000 V for 5 h, for a total of 64,000 Vh. After focusing, the individual gel strips were equilibrated for 15 min in the denaturing solution containing 6 M urea, 30% glycerol, 2% SDS, 0.375 M Tris-HCl pH 8.8, and 0.1 M DTT to reduce disulfide bonds. A second 15-min equilibration step in the same solution but containing 250 mM iodoacetamide (**IAA**) was then performed to block sulfhydryl (**SH**) groups (Goerg et al., 2000). Proteins were then subjected to the second dimension electrophoresis on 12.5% SDS polyacrylamide gels. The gels were run at 2 W per gel for 45 min, followed by 17 W per gel for 4.5 h until the dye front reached the bottom of the gel. The protein spots on analytical and preparative 2-DE gels were stained by silver and Coomassie Brilliant Blue G250, respectively. Subsequently, gel evaluation and data analysis were carried out using the ImageMaster v 7.0 program (GE Healthcare). The differences of 2-DE data were evaluated by a one-way ANOVA and a Tukey's significance test (*P* < 0.01) using SPSS 13.0 (SPSS, Chicago, IL).

### Protein Identification

**Figure 1.**
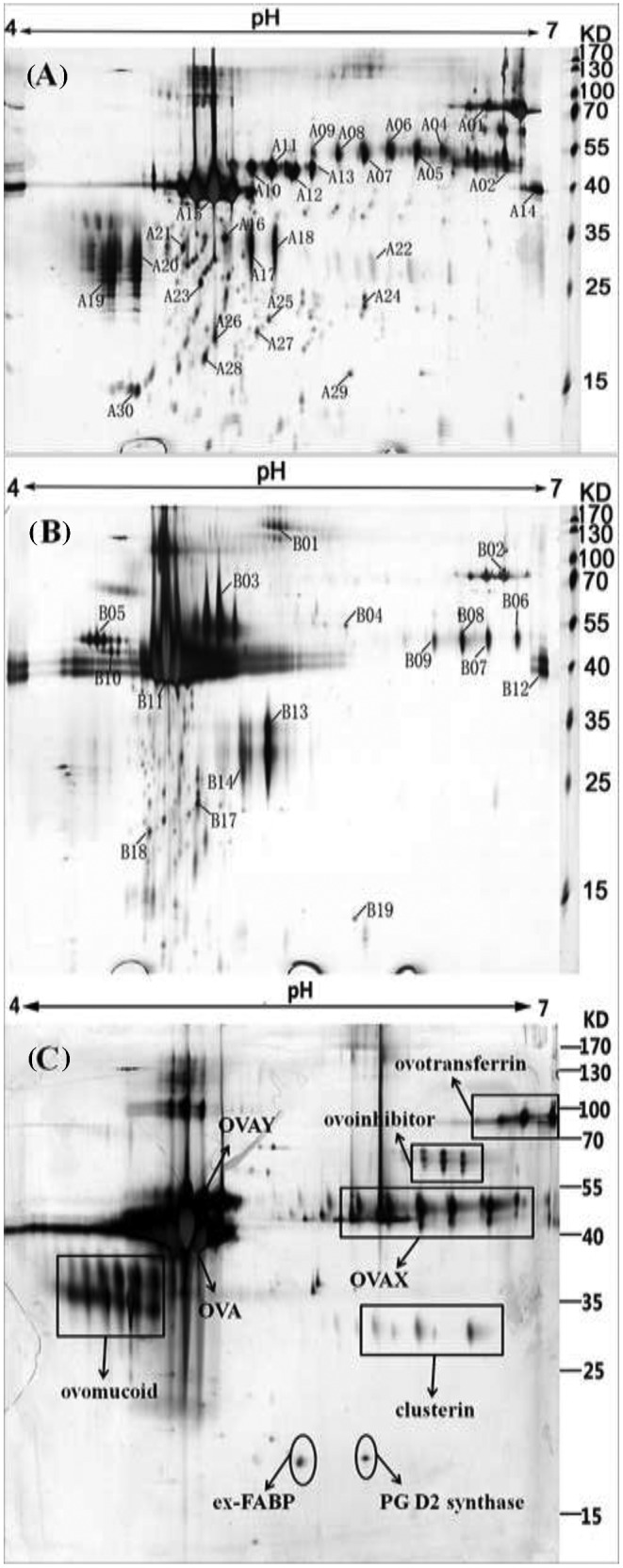
Protein pattern and identification of egg whites separated by 2-dimensional polyacrylamide gel electrophoresis (2-DE) after identifying by mass spectrometry. Spots that identified successfully were indicated by numbers and arrows. A, quail egg white; B, duck egg white; C, chicken egg white (the control).

All those with significant and reproducible changes in intensity (*P* < 0.01) were considered to be different and representative spots among the gel profiles of duck, quail, and chicken egg white. The protein spots that were identified successfully are indicated by numbers and arrows in Figure 1. The target protein spots were excised manually from 2-DE gels of quail and duck egg white, then destained, washed, and digested with sequencing-grade trypsin (Promega, Madison, WI). The samples mixed with an equivalent matrix solution (HCCA) were applied for further MALDI-TOF MS/MS analysis using a fuzzy logic feedback control system (Ultraflex MALDI-TOF-TOF mass spectrometer Bruker, Karlsruhe, Germany). Proteins were identified by searching against the nonredundant sequence database (NCBInr) via the MASCOT program (http://www.matrixscience.com). Further sequence homology analysis was conducted using the Basic Local Alignment Search Tool (BLAST).

## RESULTS AND DISCUSSION

The avian egg white proteins were separated using 2-DE as shown in Figure 1. The protein patterns of 3 avian egg whites presented obvious distinct regions (Figure 1). On one hand, comparative protein patterns of quail and duck egg white showed the obvious deficiency of the ovoinhibitor spots previously identified on chicken egg white 2-DE gel (Guerin-Dubiard et al., 2006; Qiu et al., 2012a; Qiu et al., 2012b). In addition, clusterin, ovomucoid, and prostaglandin D2 synthase (**PG D2 synthase**) were not detected in the coordinate area on the 2-DE gel and were neither identified by MALDI-TOF MS/MS analysis in duck egg white. On the other hand, 2 protein spot groups that have not been observed in a corresponding region on chicken egg white gel (one containing B05: vitellogenin-2 and another one comprising B01: deleted in malignant brain tumors 1 protein [**DMBT1**]) were duck-specific. In the case of quail, ribosomal protein L31 (**RBP L31**) (spot A18) and Lipocalin Q83 (spot A29) were specific. In addition, the rareness in quantity and scale for the ovalbumin-X spot group observed in duck egg white was another variation compared to that of chicken and quail.

In the present study, 29 protein spots representing 10 different kinds of proteins were successfully identified in quail egg white, while 17 protein spots representing 9 proteins were identified in duck egg white, respectively. These proteins were further classified into 10 protein families: Transferrin, **BPI** (Bactericidal Permeability-Increasing) Superfamily, **SERPIN** (serine proteinase inhibitor) Superfamily, Lipocalin family, Ribosomal_L31e Superfamily, KAZAL_FS Superfamily, Clusterin, **SRCR** (Scavenger Receptor Cysteine Rich) Superfamily, Vitellogenin_N, ML Superfamily (Table 1)[Table tbl1a]. Among these proteins, RBP L31 (spot A18) from quail was first identified in avian egg white, which means it was not detected in sorts of proteomic on low-abundance proteins of chicken egg white (Mann, 2007; D'Ambrosio et al., 2008; Mann and Mann, 2011). Among proteins listed in Table 1, OVAX (A04, A06, A09), OVAY (A11-A13), and RBP L31 (A18) were identified only by a single peptide (Table S1). This may render the identification somehow questionable; however, the resulting coverage may be representative enough.

**Table 1.** Protein identification by mass spectrometry (MALDI-TOF MS-MS) after 2-dimensional polyacrylamide gel electrophoresis (2-DE) analysis.

Spot	Match			Theory		Calculated		Sequence

no.^1^	to^2^	Protein name	Score^3^	Mw (Da)	pI	Mw (Da)	pI	coverage (%)
**SERPIN Superfamily**								
A14	gi|82204122	Ovalbumin [Coturnix coturnix]	98	42,467	5.29	39,574	6.84	13
A15	gi|82204122	Ovalbumin [Coturnix coturnix]	170	42,467	5.29	41,542	5.11	24
A16	gi|82204122	Ovalbumin [Coturnix coturnix]	173	41,467	5.29	34,118	5.19	20
A21	gi|82204122	Ovalbumin [Coturnix coturnix]	259	42,467	5.29	32,215	4.98	17
A23	gi|82204122	Ovalbumin [Coturnix coturnix]	174	42,467	5.29	26,100	5.07	17
A24	gi|82204122	Ovalbumin [Coturnix coturnix]	230	42,467	5.29	23,394	5.94	14
A25	gi|82204122	Ovalbumin [Coturnix coturnix]	319	42,467	5.29	21,269	5.41	14
A26	gi|82204122	Ovalbumin [Coturnix coturnix]	233	42,467	5.29	19,760	5.14	16
A27	gi|82204122	Ovalbumin [Coturnix coturnix]	282	42,467	5.29	19,903	5.35	18
A28	gi|82204122	Ovalbumin [Coturnix coturnix]	127	42,467	5.29	17,254	5.09	10
A30	gi|82204122	Ovalbumin [Coturnix coturnix]	182	42,467	5.29	14,448	4.74	6
A04	gi|448824824	ovalbumin-related protein X [Gallus gallus]	44	43,627	5.97	51,095	6.33	3
A05	gi|448824824	ovalbumin-related protein X [Gallus gallus]	52	43,627	5.97	50,892	6.21	10
A06	gi|448824824	ovalbumin-related protein X [Gallus gallus]	44	43,627	5.97	51,915	6.06	3
A07	gi|448824824	ovalbumin-related protein X [Gallus gallus]	52	43,627	5.97	50,489	5.93	10
A08	gi|448824824	ovalbumin-related protein X [Gallus gallus]	52	43,627	5.97	50,188	5.79	10
A09	gi|448824824	ovalbumin-related protein X [Gallus gallus]	44	43,627	5.97	51,095	5.65	3
A10	gi|733881415	ovalbumin-related protein Y [Meleagris gallopavo]	48	43,883	5.08	45,164	5.33	11
A11	gi|733881415	ovalbumin-related protein Y [Meleagris gallopavo]	37	43,883	5.08	45,164	5.43	3
A12	gi|733881415	ovalbumin-related protein Y [Meleagris gallopavo]	37	43,883	5.08	45,074	5.56	3
A13	gi|733881415	ovalbumin-related protein Y [Meleagris gallopavo]	37	43,883	5.08	45,164	5.64	3
**Transferrin**								
A01	gi|734703936	transferrin precursor [Meleagris gallopavo]	254	79,552	6.62	70,764	6.61	23
**BPI Superfamily**								
A02	gi|521300670	TENP protein [Gallus gallus]	95	49,014	5.89	48,518	6.67	11
**Lipocalin Superfamily**								
A17	gi|82134837	Ovoglycoprotein precursor (Alpha 1-acid glycoprotein) [Gallus gallus]	120	22,321	5.11	31,104	5.32	15
A29	gi|13641422	lipocalin Q83 [Coturnix coturnix]	210	20,368	6.75	15,777	5.85	36
**Ribosomal_L31e Superfamily**								
A18	gi|115382941	ribosomal protein L31 [Coturnix coturnix]	11	8,621	9.89	32,010	5.44	16
**KAZAL_FS Superfamily**								
A19	gi|124763	Ovomucoid [Coturnix japonica]	116	21,489	4.71	28,447	4.61	30
A20	gi|124763	Ovomucoid [Coturnix japonica]	112	21,489	4.71	28,042	4.75	14
**Clusterin**								
A22	gi|520630	Clusterin [Coturnix coturnix]	178	52,395	5.39	29,136	5.98	12
**SERPIN Superfamily**								
B03	gi|733881415	ovalbumin-related protein Y [Meleagris gallopavo]	48	43,883	5.08	64,884	5.16	8
B04	gi|733881415	ovalbumin-related protein Y [Meleagris gallopavo]	33	43,883	5.08	55,000	5.84	9
B06	gi|874487655	ovalbumin-related protein X [Anas platyrhynchos]	83	44,185	6.29	49,583	6.78	28
B07	gi|874487655	ovalbumin-related protein X [Anas platyrhynchos]	72	44,185	6.29	49,339	6.62	11
B08	gi|874487655	ovalbumin-related protein X [Anas platyrhynchos]	73	44,185	6.29	48,494	6.48	20
B09	gi|874487655	ovalbumin-related protein X [Anas platyrhynchos]	75	44,185	6.29	48,614	6.33	25
B10	gi|906366229	Ovalbumin [Anas platyrhynchos]	84	43,396	4.96	44,261	4.58	26
B12	gi|906366229	Ovalbumin [Anas platyrhynchos]	216	43,595	4.96	39,884	6.92	40
B17	gi|906366229	Ovalbumin [Anas platyrhynchos]	61.6	43,396	4.96	23,236	5.05	14
B18	gi|906366229	Ovalbumin [Anas platyrhynchos]	40.5	43,396	4.96	21,189	4.87	6
**SRCR Superfamily**								
B01	gi|483510368	Deleted in malignant brain tumors 1 protein [Anas platyrhynchos]	168	54,074	4.53	153,334	5.49	20
**Transferrin**								
B02	gi|514782616	Serotransferrin [Anas platyrhynchos]	411	79,064	6.40	76,083	6.72	36
**Vitellogenin_N**								
B05	gi|483509788	Vitellogenin-2, partial [Anas platyrhynchos]	141	186,092	8.40	49,339	4.50	6
**BPI Superfamily**								
B11	gi|541049931	TENP protein, partial [Anas platyrhynchos]	60	38,566	4.55	45,704	4.87	30
**Lipocalin Superfamily**								
B13	gi|514739030	alpha-1-acid glycoprotein-like, partial [Anas platyrhynchos]	286	15,295	6.06	29,856	5.43	38
B14	gi|514739030	alpha-1-acid glycoprotein-like, partial [Anas platyrhynchos]	142	15,295	6.06	29,535	5.29	43

**Table 1 tbl1a:** continued.

Spot	Match			Theory		Calculated		Sequence

no.^1^	to^2^	Protein name	Score^3^	Mw (Da)	pI	Mw (Da)	pI	coverage (%)
**ML super family**								
B19	gi|483523530	Lymphocyte antigen 86, partial [Anas platyrhynchos]	360	18,206	5.75	13,129	5.89	58

^1^Spot ID represents the protein spot number on the 2-dimensional gel electrophoresis gel image.

^2^Accession numbers of matched proteins according to the National Center for Biotechnology Information nonredundant sequence (NCBInr) database.

^3^MASCOT score.

A large amount of the identified protein spots from quail and duck egg white were designated to the ovalbumin family, which is composed of ovalbumin (**OVA**), ovalbumin-related protein Y (**OVAY**), and ovalbumin-related protein X (**OVAX**). These 3 homologous genes in chicken genome (*Gallus gallus*) are located within a 46 kb locus on chromosome 2. Although with high similarity in their primary sequences, these 3 homologs exhibit distinct and subtle physicochemical and structural differences, which might be associated with specific functions (Da Silva et al., 2015). Although classified in the SERPIN family, ovabumin was proven to have no protease inhibitory activity in its native form (Hunt and Dayhoff, 1980). Meanwhile, the physiological function of OVAY is not known but recent data have revealed that it was the major differentiated protein between fertilized and unfertilized chicken egg whites, which indicated a possible pivotal role in early embryonic development (Qiu et al., 2013; Da Silva et al., 2015). OVAX was suggested to play a role in egg defense as it exhibits antimicrobial activities against at least 2 pathogens, *Listeria monocytogenes* and *Salmonella enterica Enteritidis* via its heparin-binding domain (Rehault-Godbert et al., 2013). Six spots (A04-A09) as well as 4 spots (B06-B09) were detected as OVAX in quail and duck egg white, respectively (Figure 1 and Table 1). These identified spots showed higher molecular weight (**MW**) than the theoretical ones, suggesting the possible glycosylation. Meanwhile, it was found that duck OVAX showed less in spot quantity and scale and was located in the relatively more alkaline region compared to that of quail and chicken (Figure 1). Compared to the 4 major OVAY spots (A10-A13) from quail, the chicken-OVAY and duck-OVAY major spots are more concentrated (Figure 1). Moreover, the MW of the chicken-OVAY and duck-OVAY were significantly higher than quail-OVAY. The polymorphism of ovalbumin Y has been reported and suggested to be formed by alternative splicing processes but not by various phosphorylation or glycosylation levels nor by genetic variations (Nau et al., 2005). Thus, the dispersive separating OVAY spots of quail egg white may indicate more distinct polymorphism from alternative splicing. Ten protein spots (A15, A16, A21, A23-28, and A30) were identified as quail ovalbumin, most of which showed lower apparent MW than the theoretical value. Such ovalbumin spots were commonly observed during chicken egg storage, which were suggested as degraded ovalbumin fragments (Omana et al., 2011; Qiu et al., 2012a; Qiu et al., 2012b). As the fresh laid poultry eggs were collected and used in this study, the larger amount of spliced ovalbumin spots may demonstrate a higher proteolytic degradation in quail egg white soon after laid. Noticeably, spot A14 of quail and B12 from duck appearing in the alkaline region (pI ∼7) were detected as ovalbumin. It still needs to be verified whether these spots were contaminations or in combination to alkaline proteins (eg., lysozyme). According to Sun and Hayakawa (2002), duck ovalbumin possesses a larger number of disulfide bonds than its chicken counterpart does, leading to clear differences in the translucency and viscoelasticity of heat-induced egg white gel between duck and chicken.

Ovomucoid is one of the major proteins found in chicken egg white. Also, it is well known as a “trypsin inhibitor” and is considered to be the main food allergen in egg white (Abeyrathne et al., 2015). One of the most astonishing findings in this study is the deficiency of ovomucoid spots on the duck egg white 2-DE gel (Figure 1B), as former studies have suggested a high concentration of ovomucoid in duck albumen (Miguel et al., 2005). Instead, 2 major protein spots with high intensity (B13 and B14) from duck were detected as ovoglycoprotein (alpha-1-acid glycoprotein-like, partial (*Anas platyrhynchos*), gi: 514739030) and was also identified in quail at coordinate position on the gel (A17 and A18), but was not found in chicken. A previous chicken egg white proteomic study based on 2-DE demonstrated that ovomucoid spots contained ovoglycoprotein and riboflavin-binding protein, suggesting possible mixture of these 3 proteins (Guerin-Dubiard et al., 2006). Thus, ovomucoid may also exist in spot B13 and B14 but failed to be detected by MALDI-TOF MS/MS analysis in the present study. Even though, the discrepancy shown on the comparative 2-DE map implied the differentiated physicochemical characteristics of ovomucoid (if it does exist) in duck egg white. The alpha-1-acid glycoprotein-like of *Anas platyrhynchos* showed 63% identity to chicken alpha-1-acid glycoprotein in the amino acid level. The experimental MW (30 kDa) is twice that of the theoretical one (15 kDa), supposed as its dimmer. Numerous activities of potential physiological significance of AGP have been reported including various immunomodulating effects, the ability to bind and to carry numerous basic and neutral lipophilic drugs from endogenous and exogenous origin (Fournier et al., 2000). Clusterin, which was supposed to be related to the egg white thinning during storage (Omana et al., 2011; Qiu et al., 2012b), was not detected in duck egg white either. Other proteins/mechanism should exist for duck to maintain the stabilization of egg white proteins to prevent their aggregation and precipitation during storage. Additionally, 2 proteins with relatively low MW, extracellular fatty acid-binding protein precursor (**ex-FABP**) and PG D2 synthase (Figure 1C), were absent from duck egg white. Whereas in the corresponding region, a protein spot (B19) was depicted as Lymphocyte antigen 86 (also named as myeloid differentiation factor 1, MD-1). *Anas platyrhynchos* MD-1 showed 84% identity to its homolog of *Gallu gallus*, which was first identified in chicken egg white by using 1-D PAGE and LC-MS/MS and MS^3^ (Mann, 2007). Lymphocyte antigen 86 belongs to the ML superfamily, which includes multiple members of unknown function in animals and plants. Some of these ML proteins regulate lipid metabolism, the host response to pathogen components such as lipopolysaccharides (**LPS**), and other cellular functions involving lipid recognition (Inohara and Nunez, 2002). Comparatively, serotransferrin, which amino acid sequences show high homology (82%) to chicken's ovotransferrin, was observed with relatively lower abundance in duck egg white (B02) than those of quail (A01) and chicken (Figure 1), which was in good agreement with the previously reported result (Miguel et al., 2005).

Although several well-known egg white proteins were absent from the 2-DE map of duck egg white, 2 protein spots (B01 and B05) were identified to be specific. Spot B01 was designated as DMBT1, which belongs to the SRCR protein family. *Anas platyrhynchos* DMBT1 (gi: 483510368) showed higher identity (87%) to *Gallus gallus* PIT54 protein precursor (gi: 46395491) than its homolog (gi: 513233654, identity: 54%) in *Gallus gallus*. Both DMBT1 and PIT54 proteins have 4 repeats of SRCR domain, which plays an important role in innate immunity by directly binding to a broad range of pathogens including bacteria and HIV (Ligtenberg et al., 2010; Madsen et al., 2010) or interacting with various other defense factors (Ligtenberg et al., 2007). Furthermore, the important role of mammal DMBT1 has been demonstrated in the process of fertilization (Ambruosi et al., 2013). Spot B05 was identified as vitellogenin-2, which is a typical major yolk component. The detection of vitellogenin-2 in duck egg white could not be simply explained as contaminant as it was also included in the chicken egg white proteome analysis (Mann and Mann, 2011). According to Mann and Mann (2011), vitellogenin-2 could be liberated from the ovary together with the egg and migrated into the oviduct, mixing with egg white proteins secreted in the magnum compartment (Mann and Mann, 2011). It has been revealed that vitellogenin-derived proteins participate in the transport mechanisms for the movement of lipid, phosphorus, and metals to the yolk and serve directly as nutrient sources during avian embryonic development (Wang et al., 1983). It is noteworthy that these 2 major protein spots (B01 and B05) unique in duck egg white might infer the different mechanisms for cell defense and nutrition transport during duck reproduction.

In this study, RBP L31 (spot A18) was detected from quail egg white, which is a part of the 60S large ribosomal subunit. This protein was identified for the first time in avian egg white. It has been reported that egg-ribosomal proteins appeared to undergo rapid changes after fertilization, which may be causally related to the acceleration of protein synthesis following fertilization (Takeshima and Nakano, 1983). Another *Coturnix coturnix* specific protein was identified as Lipocalin Q83 (spot A29), which is a lipocalin family member. Quail Lipocalin Q83 displays a high degree of sequence identity with a developmentally regulated chicken protein (termed Ch21 or ex-FABP), which plays a pivotal biological role in cellular growth and development (Hartl et al., 2003). A recent study also demonstrated that quail Lipocalin Q83 is a siderocalin that participates in the innate immune response by interfering with bacterial siderophore-mediated iron uptake, and is involved in several physiological and pathological processes such as inflammation, iron delivery, tissue differentiation, and cancer progression (Coudevylle et al., 2010; Coudevylle et al., 2011). Moreover, 2 BPI protein family members, transiently expressed in neural precursors (**TENP**) protein (A02, gi: 521300670) and TENP protein, partial (B11, gi: 541049931), were identified from quail and duck respectively. The BPI family consists of a series of antimicrobial proteins that bind to LPS from the outer membrane of Gram-negative bacteria. The detection of different kinds of BPI family members in these avian egg whites might indicate distinct defense mechanisms evolved under various environments.

In conclusion, qualitative and quantitative differences of egg white proteins were observed among poultry species involved in this study. Ovoinhibitor was observed as a major protein specific to *Gallus gallus*. Comparatively, the quail egg white proteome pattern seems closer related with that of chicken, which both belong to the Galliformes order. Whereas the duck (belonging to the Anseriformes order) egg white protein distribution on the 2-DE map was more diverse. A series of major proteins, including ovoinhibitor, ovomucoid, clusterin, ex-FABP, and PG D2 synthase was not detected from duck egg white, while 2 major protein spots identified as DMBT1 and vitellogenin-2 were found specific to duck in corresponding ranges on the 2-DE map. In quail egg white, RBP L31 (spot A18) and Lipocalin Q83 (spot A29) were found specific, while ovoinhibitor and PG D2 synthase were not detected. These diversities may indicate the unique needs for egg white protein functions (such as cell defense or regulating/supporting embryonic development) due to the different inhabiting environments or reproduction demand during long-term evolution. This interspecies variability also gives insight into the advantages involved in application on egg white proteins from various egg sources, which may present novel beneficial properties in the food industry or related to human health. Based on the 2-DE method and without the elimination of high-abundance proteins, only a few egg white proteins from quail and duck were separated and identified in the present study, which should contain only a small part of the total egg white proteins. Therefore, further studies are required to identify and characterize more novel proteins by using latest proteomic techniques.

## SUPPLEMENTARY DATA

**Table S1.** Matched peptide sequences of each protein identified through mass spectrometry Supplementary data is available at PSA Journal online.
